# Maintenance Therapy with FOLFIRI after FOLFIRINOX for Advanced Pancreatic Ductal Adenocarcinoma: A Retrospective Single-Center Analysis

**DOI:** 10.1155/2019/5832309

**Published:** 2019-12-06

**Authors:** Caspar Franck, Ali Canbay, Peter Malfertheiner, Marino Venerito

**Affiliations:** Otto-von-Guericke University Hospital, Department of Gastroenterology, Hepatology and Infectious Diseases, Leipziger Str. 44, 39120 Magdeburg, Germany

## Abstract

**Objectives:**

Patients with pancreatic ductal adenocarcinoma (PDA) receiving FOLFIRINOX often develop oxaliplatin-induced polyneuropathy, which limits the continuation of this therapy. We evaluated the efficacy and safety of FOLFIRI maintenance treatment after FOLFIRINOX induction in a retrospective single-center study.

**Methods:**

Patients with advanced PDA treated with FOLFIRI as maintenance therapy after achieving disease control under FOLFIRINOX according to the local operating procedure between 2011 and 2016 were identified. Medical records of this group were evaluated retrospectively.

**Results:**

Overall, 22 patients with PDA were treated with FOLFIRI (mean age 59 years, 55% female, 45% male). Before receiving FOLFIRI, all patients were treated with FOLFIRINOX for a median of 4 months. The median progression-free survival (PFS) under FOLFIRI maintenance therapy was 8 months. Side effects grade 3-4 (CTCAE v4.0) were observed in 18% of patients receiving FOLFIRI. Considering together FOLFIRINOX induction and subsequent FOLFIRI maintenance therapy, the median PFS was 11 months. The median overall survival (OS) from the beginning of palliative treatment was estimated at 46 months.

**Conclusions:**

In the selected group of PDA patients achieving disease control with FOLFIRINOX, FOLFIRI maintenance therapy was feasible, safe, and effective, with some patients achieving long-term disease stabilization.

## 1. Introduction

Pancreatic ductal adenocarcinoma (PDA) is a substantial health concern and socioeconomic burden responsible for the loss of nearly one million quality-adjusted life-years (QALYs) per year in Europe [1]. In 2017, PDA has overtaken breast cancer in the EU as a third leading cause of cancer-related death and is estimated to become the second most common cause of cancer-related mortality in 2030 [2, 3]. Despite a considerable improvement of the 5-year survival rate reached during the past decades for most malignancies, the advancements for PDA (5-year survival: 2% in 1975 to 6% in 2009) were less remarkable [4]. The prognosis of PDA is still very poor: only 10–20% of patients are diagnosed at an early stage to receive curative intended resection and most of them experience recurrence following pancreaticoduodenectomy (PD) [5].

Since 2011, the FOLFIRINOX regimen is recommended and widely used as first-line treatment for patients with inoperable PDA in good performance status (ECOG score 0-1) [6–9]. With FOLFIRINOX, a median progression-free survival (PFS) of 6.4 months and improvement of life quality in comparison to single-agent treatment with gemcitabine was proven [9, 10]. However, treatment with FOLFIRINOX is associated with high rates of grade 3/4 toxicities including neutropenia (46%), diarrhea (13%), and sensory neuropathy (9%) [9]. According to our experience, neutropenia and diarrhea are usually manageable, whereas oxaliplatin-induced polyneuropathy often precludes the further administration of FOLFIRINOX.

In our GI-oncology department, early withdrawal of oxaliplatin and continuation of FOLFIRI represents a standard strategy for PDA patients with increasing oxaliplatin-induced polyneuropathy achieving disease control with FOLFIRINOX.

To the best of our knowledge, this is the first report on efficacy and safety of FOLFIRI as a maintenance treatment for PDA patients after induction chemotherapy with FOLFIRINOX.

## 2. Materials and Methods

**Figure 1:**
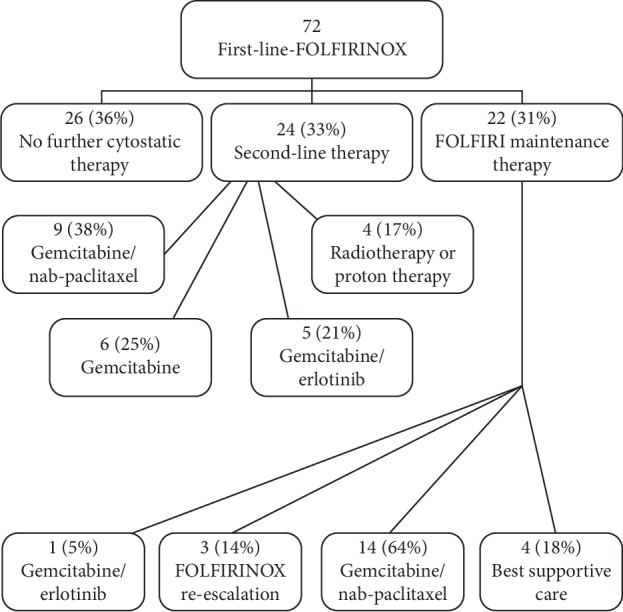
Patient flow chart (*n* = 72).

Between January 2011 and December 2016, 72 patients with histologically confirmed, nonresectable PDA were treated with FOLFIRINOX (oxaliplatin 85 mg/m^2^ d1, irinotecan 180 mg/m^2^, leucovorin 400 mg/m^2^, 5-fluorouracil 400 mg/m^2^, 5-fluorouracil 2400 mg/m^2^ 46 h; qd15) at our site. According to our therapeutic standard, concomitant supportive medication with dexamethasone 8 mg, palonosetron 0.25 mg and atropine 0.25 mg was administered. Furthermore, most patients (86%) received a prophylactic administration of recombinant long acting (pegylated) granulocyte-colony stimulating factor (G-CSF). Second-line or maintenance therapy could be performed in 46 of 72 patients (64%). Following disease progression, common second-line regimens were gemcitabine/nab-paclitaxel, gemcitabine alone, and gemcitabine/erlotinib (Figure 1). In some patients, additional individualized strategies such as radiotherapy or proton therapy were performed. In 22 patients achieving disease control on FOLFIRINOX, oxaliplatin was withdrawn at the individual physician's choice due to increasing peripheral neurotoxicity. Subsequently, FOLFIRI (irinotecan 180 mg/m^2^ d1, leucovorin 400 mg/m^2^, 5-fluorouracil 400 mg/m^2^ bolus, 5-fluorouracil 2400 mg/m^2^ 46 h; qd15) was continued until tumor progression. Records of all the 22 patients receiving FOLFIRI maintenance therapy at our center were retrieved and reviewed retrospectively. Therapy response was assessed by contrast-enhanced CT-scans every 3 months and rated according to the RECIST v1.1 criteria. These treatment data were last updated in August 2018. In addition, missing survival data of the included patients were obtained from the clinical cancer registry of Saxony-Anhalt. However, due to a software actualization, the last update for the dates of death was possible in November 2017. All data were entered, analyzed, and represented graphically using Microsoft Excel®. The survival data were estimated using the Kaplan–Meier method. Patients were censored at the time of last follow-up. Therapy-associated adverse events were assessed in accordance with the current CTCAE v4.0 guidelines [11].

## 3. Results

**Figure 2:**
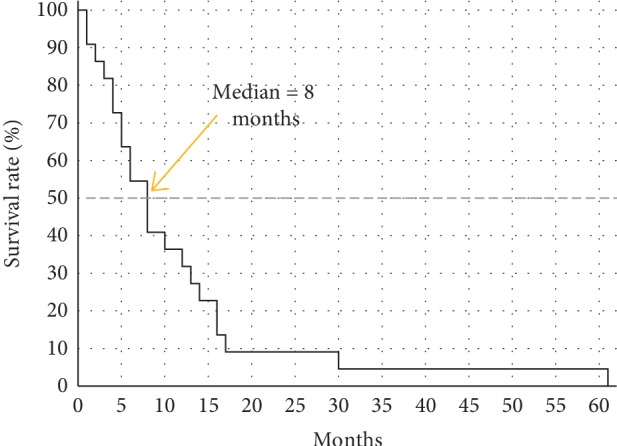
Progression-free survival under FOLFIRI (*n* = 22, range 1–61 months).

**Figure 3:**
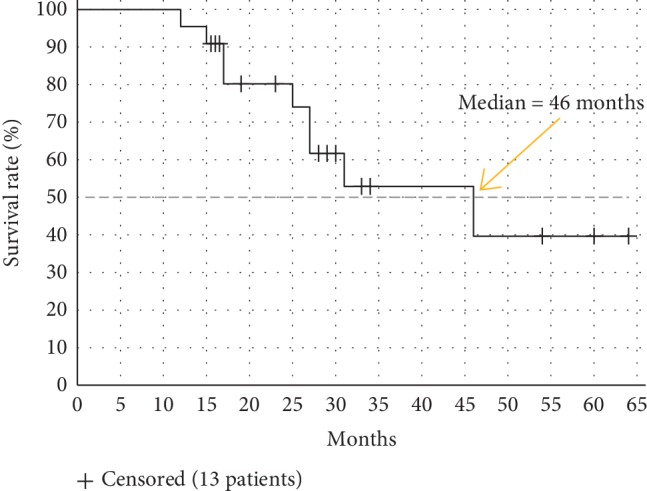
Overall survival after 1 cycle of FOLFIRINOX (*n* = 22).

Mean age at diagnosis of PDA was 59 years (range 37 to 74 years, median 60.5 years). There were 12 (55%) female and 10 (45%) male study participants (Table 1). All patients were in good general condition. At therapy start, 77% were rated as ECOG performance score 0 and 23% as 1. Most patients (82%) had at least one chronic comorbidity. Among them, arterial hypertension with need of drug treatment was the most common (55%). One female patient had HNPCC syndrome with history of ovarian carcinoma. The most common indication for FOLFIRINOX chemotherapy was a primarily metastatic PDA (55%), whereas the proportion of patients with locally advanced disease, local recurrence, and systemic relapse after PD was 14%, 23%, and 9%, respectively. The predominant primary tumor localization was the pancreatic head (50%). Oxaliplatin was discontinued after a median of 4 months (range 2 to 6 months), mostly because of increasing peripheral neuropathy (Table 2). Two patients developed grade 3 polyneuropathy. All patients received a minimum of 5 and a maximum of 12 cycles FOLFIRINOX. Before switching to maintenance therapy, 5 patients (23%) showed stable disease, 16 (73%) had partial response, and 1 patient (5%) complete response according to the RECIST v1.1 criteria. The median progression-free survival (PFS) under FOLFIRI therapy was 8 months (Figure 2). The longest PFS was observed in a 76-year-old woman with continuous FOLFIRI treatment in the past 61 months (Table 3). The median PFS under FOLFIRINOX and subsequent FOLFIRI maintenance was 11 months. Overall, 4 (18%) patients developed adverse events higher than grade 2 on FOLFIRI. Hematologic side effects were the most common with both neutropenia and anemia in 14% of the patients. In 6 patients, a protocol modification with dose reduction to 75% or interval prolongation to 21 days was necessary. In half of the patients receiving FOLFIRI, G-CSF therapy was still necessary in order to avoid delays in the chemotherapy schedule. All included patients experienced progressive disease over time. The most common regimen following FOLFIRI was gemcitabine/nab-paclitaxel (64%, Table 3). The median overall survival (OS) from the beginning of the palliative treatment was 46 months (Figure 3). The majority of patients included in the present analysis (59%) are still alive.

## 4. Discussion

Herein, we provide for the first time data on safety and efficacy of FOLFIRI maintenance therapy in patients with advanced PDA achieving disease control with the FOLFIRINOX regimen. Therapeutic options for patients with nonresectable PDA are limited. According to the current guidelines, both FOLFIRINOX and gemcitabine/nab-paclitaxel represent effective first-line options for patients with good performance status (ECOG score 0-1) [6, 7]. However, there are no studies comparing these regimens and no objective criteria for an individual selection of chemotherapy are available. Thus, the choice of the first-line regimen and of the dose-intensity to administer is often made based on patients' age, general condition, and comorbidities as well as the side-effect profile of the regimens.

For second-line therapy of PDA, the data are even more scarce. In Europe, after a gemcitabine-based first-line therapy, the NAPOLI regimen (nal-irinotecan 80 mg/m^2^; leucovorin 400 mg/m^2^; 5-fluorouracil 2400 mg/m^2^ 46 h; qd15) is approved for second-line treatment [12]. With respect to patients with progressive disease after a fluoropyrimidine-based therapy (i.e., FOLFIRINOX), no approved regimens are available. Due to the lack of options in this indication, gemcitabine/nab-paclitaxel is often offered as second-line treatment (off-label use) to patients with still good performance status.

Published experience with FOLFIRI maintenance therapy after FOLFIRINOX for PDA is limited to a case report describing long-term response to FOLFIRI in a patient with metastatic PDA [13]. Thus far, no recommendation has been generated from the current guidelines on how to treat patients with disease control on first-line FOLFIRINOX [6–8]. In clinical practice, FOLFIRINOX is usually administered until tumor progression or appearance of compromising side effects. Neurotoxicity is the most frequent dose-limiting toxicity of oxaliplatin, leading to an often irreversible damage of peripheral nerve fibers which may even aggravate after withdrawal of the drug [14]. Thus, a continuous monitoring of polyneuropathy, weighting beneficial cytostatic activity, and side effects is mandatory.

Data concerning maintenance therapy in patients with metastatic PDA achieving disease control on FOLFIRINOX are limited to 2 French studies [15, 16] and the POLO trial. In the first French retrospective study, patients with PDA (*n* = 30) and disease control after 4 to 8 cycles of FOLFIRINOX were switched to oral capecitabine (2,000–2,500 mg/m^2^ d1-14; qd22). Safety of capecitabine was generally good with hand-foot syndrome being the most relevant adverse event (16.6% > grade 2). The median interval to tumor progression (PFS) on capecitabine maintenance therapy was 5 months. The second French study, the PRODIGE 35/PANOPTIMOX phase II randomized trial, investigating maintenance therapy for metastatic PDA after FOLFIRINOX induction, was presented on the ASCO annual meeting of 2018 and is still not fully published [16]. Patients were randomized to receive FOLFIRINOX over 6 months (arm A, *n* = 91) or FOLFIRINOX induction for 8 cycles (4 months) followed by 5-fluorouracil/leucovorin maintenance therapy (leucovorin 400 mg/m^2^ d1, 5-fluorouracil 400 mg/m^2^, 5-fluorouracil 2400 mg/m^2^ 46 h; qd15) and FOLFIRINOX reinduction at disease progression (arm B, *n* = 92). No relevant difference in PFS and OS was observed between arm A and B. Interestingly, an even higher rate of severe neurotoxicity (>grade 2) was observed in arm B (19% vs. 10%). The median time of tumor control (PFS) on 5-fluorouracil/leucovorin after FOLFIRINOX induction was 3.3 months. Due to the limited PFS observed in the PRODIGE 35/PANOPTIMOX study for patients on maintenance therapy with 5-fluorouracil/leucovorin, an early reescalation to FOLFIRINOX was often necessary, leading to both a higher cumulative oxaliplatin dose and a higher rate of severe neurotoxicity. Compared to the published data on capecitabine and 5-fluorouracil/leucovorin maintenance therapy, a longer median PFS (8 months) was observed in our patients receiving FOLFIRI. This effect may be attributable to the 3-drug chemotherapy without renouncing to the topoisomerase inhibitor.

The recently published randomized, placebo-controlled, phase 3 POLO trial evaluated the efficacy of olaparib as maintenance therapy in the selected subgroup of patients who had a germline *BRCA1* or *BRCA2* mutation and metastatic PDA and disease that had not progressed during first-line platinum-based chemotherapy [17]. BRCA mutations predict *inter alia* an improved treatment response to platin containing chemotherapy [18]. Germline *BRCA1* or *BRCA2* mutations were detected in 7.5% of the screened patients with metastatic PDA. More than 80% of study participants received FOLFIRINOX variants before randomization. The median PFS for patients receiving olaparib was significantly longer than in the placebo group (7.4 months vs. 3.8 months) and similar to that observed in our patients receiving FOLFIRI. However, with respect to OS, an interim analysis, at a data maturity of 46%, showed no difference between the olaparib and placebo groups (median, 18.9 months vs. 18.1 months).

A similar median overall survival (17 months) was observed in the study investigating capecitabine maintenance therapy. In our cohort, a median OS of 46 months was estimated according to Kaplan–Meier. The reliability of our data is limited because the majority of the studied patients are still living and therefore censored in the survival estimation (Figure 3). However, even assuming that all censored patients had died at time of the analysis, the median OS would be 26.5 months, which is quite longer than that in the capecitabine maintenance therapy study.

One may argue that the survival benefit seen in our study is related to the inclusion of patients with locally advanced disease which have presumably a better prognosis compared to those with distant metastases [19]. However, similar PFS was observed between the groups with locally advanced and metastatic disease (9 vs. 7 months).

According to our retrospective evaluation, FOLFIRI maintenance therapy is feasible, safe, and effective, with some PDA patients achieving disease control for a very long period. Thus, it is worth testing FOLFIRI maintenance therapy in a prospective trial. To identify patients who benefit the most from the maintenance therapy with FOLFIRI, the trial design should include a comprehensive biomarker program in order to assess the association of biomarkers with efficacy outcomes.

## Figures and Tables

**Table 1:** Characteristics of study participants (*n* = 22).

Age (years)	
Mean	59.0
Median	60.5
Range	37–74

Sex (no./%)	
Female	12/55%
Male	10/45%

ECOG performance status score (no./%)	
0	17/77%
1	5/23%

Pancreatic tumor location (no./%)	
Head	11/50%
Body	7/32%
Tail	4/18%

Biliary stent (no./%)	
Yes	3/14%
No	19/86%

Pancreaticoduodenectomy (no./%)	
Yes	7/32%
No	15/68%

Indication for cytostatic therapy (no./%)	
Primary locally inoperable	3/14%
Primary metastasized	12/55%
Local recurrence after PD	5/23%
Metastatic recurrence after PD	2/9%

Known chronic diseases (no./%)	
Medically treated hypertension	12/55%
Diabetes type II	6/27%
COPD	3/14%
Renal insufficiency > WHO I°	4/18%

**Table 2:** Characteristics of FOLFIRINOX treatment.

Time on FOLFIRINOX (months)	
Median	4
Range	2–6

Response (no./%)	
Complete response	1/5%
Partial response	16/73%
Stable disease	5/23%

Adverse events (no./%)	
Peripheral neuropathy	22/100%
Peripheral neuropathy > grade 2	2/9%
Nausea and vomiting	10/45%
Nausea and vomiting > grade 2	0/0%
Diarrhea	3/14%
Diarrhea > grade 2	0/0%
Neutropenia	5/23%
Neutropenia > grade 2	3/14%
Thrombocytopenia	2/9%
Thrombocytopenia > grade 2	1/5%
Anemia	1/5%
Anemia > grade 2	0/0%

**Table 3:** Characteristics of FOLFIRI treatment.

Time on FOLFIRI (months)	
Median	8
Range	1–61

Protocol modification	
Dose reduction (75%)	4/18%
Interval prolongation (qd21)	2/9%

Adverse events (no./%)	
Peripheral neuropathy	21/95%
Peripheral neuropathy > grade 2	1/5%
Nausea and vomiting	16/73%
Nausea and vomiting > grade 2	0/0%
Diarrhea	5/23%
Diarrhea > grade 2	0/0%
Neutropenia	11/50%
Neutropenia > grade 2	3/14%
Thrombocytopenia	1/5%
Thrombocytopenia > grade 2	0/0%
Anemia	3/14%
Anemia > grade 2	0/0%

Treatment after disease progress (no./%)	
Gemcitabine/NAB-paclitaxel	14/64%
Gemcitabine/erlotinib	1/5%
FOLFIRINOX re-escalation	3/14%
Best supportive care	4/18%

## Data Availability

The data used to support the findings are tables and figures included within the work.

## References

[1] Carrato A., Falcone A., Ducreux M. (2015). A systematic review of the burden of pancreatic cancer in Europe: real-world impact on survival, quality of life and costs. *Journal of Gastrointestinal Cancer*.

[2] Rahib L., Smith B. D., Aizenberg R., Rosenzweig A. B., Fleshman J. M., Matrisian L. M. (2014). Projecting cancer incidence and deaths to 2030: the unexpected burden of thyroid, liver, and pancreas cancers in the United States. *Cancer Research*.

[3] Ferlay J., Partensky C., Bray F. (2016). More deaths from pancreatic cancer than breast cancer in the EU by 2017. *Acta Oncologica*.

[4] Siegel R., Ma J., Zou Z., Jemal A. (2014). Cancer statistics, 2014. *CA: A Cancer Journal for Clinicians*.

[5] Gillen S., Schuster T., Büschenfelde C. M. Z., Friess H., Kleeff J. (2010). Preoperative/neoadjuvant therapy in pancreatic cancer: a systematic review and meta-analysis of response and resection percentages. *PLoS Medicine*.

[6] Ducreux M., Cuhna A. S., Caramella C. (2015). Cancer of the pancreas: ESMO Clinical Practice Guidelines for diagnosis, treatment and follow-up. *Annals of Oncology*.

[7] Tempero M., Malafa M., Al-Hawary M. (2016). *NCCN Clinical Practice Guidelines In Oncology—Pancreatic Adenocarcinoma*.

[8] Seufferlein T., Porzner M., Becker T. (2013). S3-guideline exocrine pancreatic cancer. *Zeitschrift für Gastroenterologie*.

[9] Conroy T., Desseigne F., Ychou M. (2011). FOLFIRINOX versus gemcitabine for metastatic pancreatic cancer. *New England Journal of Medicine*.

[10] Lambert A., Jarlier M., Bourgade S. G., Conroy T. (2017). Response to FOLFIRINOX by gender in patients with metastatic pancreatic cancer: results from the PRODIGE 4/ACCORD 11 randomized trial. *PLoS One*.

[11] National Institute of Cancer (2010). *Common Terminology Criteria for Adverse Events (CTCAE) Version 4.0*.

[12] Wang-Gillam A., Li C.-P., Bodoky G. (2016). Nanoliposomal irinotecan with fluorouracil and folinic acid in metastatic pancreatic cancer after previous gemcitabine-based therapy (NAPOLI-1): a global, randomised, open-label, phase 3 trial. *The Lancet*.

[13] Nikolaou C., Matikas A., Papavasilopoulou M., Mavroudis D., Vamvakas L. (2015). Prolonged complete response in a patient with metastatic pancreatic adenocarcinoma after FOLFIRINOX chemotherapy and maintenance with FOLFIRI. *Case Reports in Oncological Medicine*.

[14] Burakgazi A. Z., Messersmith W., Vaidya D., Hauer P., Hoke A., Polydefkis M. (2011). Longitudinal assessment of oxaliplatin-induced neuropathy. *Neurology*.

[15] Reure J., Follana P., Gal J. (2016). Effectiveness and tolerability of maintenance capecitabine administrated to patients with metastatic pancreatic cancer treated with first-line FOLFIRINOX. *Oncology*.

[16] Dahan L., Phelip J. M., Malicot K. L. (2018). FOLFIRINOX until progression, FOLFIRINOX with maintenance treatment, or sequential treatment with gemcitabine and FOLFIRI.3 for first-line treatment of metastatic pancreatic cancer: a randomized phase II trial (PRODIGE 35-PANOPTIM). *Journal of Clinical Oncology*.

[17] Golan T., Hammel P., Reni M. (2019). Maintenance olaparib for germline BRCA-mutated metastatic pancreatic cancer. *New England Journal of Medicine*.

[18] Golan T., Kanji Z. S., Epelbaum R. (2014). Overall survival and clinical characteristics of pancreatic cancer in BRCA mutation carriers. *British Journal of Cancer*.

[19] Suker M., Beumer B. R., Sadot E. (2016). FOLFIRINOX for locally advanced pancreatic cancer: a systematic review and patient-level meta-analysis. *The Lancet Oncology*.

